# Genetic biomarkers and individual susceptibility in healthcare workers exposed to waste anesthetic gases: a systematic review and meta-analysis

**DOI:** 10.3389/fmed.2026.1845483

**Published:** 2026-05-08

**Authors:** Bin Li, Huadong Zhu, Yueping Ge

**Affiliations:** 1 Operating Theatre, Tongde Hospital of Zhejiang Province, Hangzhou, China; 2 Department of Anesthesiology, Tongde Hospital of Zhejiang Province, Hangzhou, China

**Keywords:** genotoxicity biomarkers, healthcare workers, occupational exposure, precision medicine, waste anesthetic gases

## Abstract

**Background:**

From a precision medicine perspective, this systematic review and meta-analysis synthesizes evidence on the genotoxic effects of waste anesthetic gases (WAGs) in healthcare workers. It aims to identify robust biomarkers for individualized risk assessment and to clarify the pattern of DNA damage to inform targeted monitoring strategies.

**Methods:**

We conducted a comprehensive search of four databases up to September 2025, following PRISMA 2020 guidelines. Observational studies comparing genotoxic biomarkers including comet assay parameters, micronucleus frequency and chromosomal aberrations, between WAG-exposed and non-exposed healthcare workers were included. Pooled mean differences (MD) with 95% confidence intervals (CI) were calculated using random- or fixed-effects models. Study quality was assessed using the Newcastle-Ottawa Scale.

**Results:**

Thirty-three studies were included. Occupational exposure to WAGs (e.g., nitrous oxide, isoflurane, sevoflurane) was associated with significantly increased DNA damage, measured by comet assay scores (MD = 7.58, 95% CI = 5.29–9.86), tail length (MD = 4.21, 95% CI = 0.26–8.15), and %tail DNA (MD = 2.97, 95% CI = 1.24–4.70). Micronucleus frequency was also elevated in both buccal cells (MD = 0.24 per 1,000 cells, 95% CI = 0.13–0.35) and lymphocytes (MD = 3.74 per 1,000 cells, 95% CI = 3.33–4.14). In contrast, the level of total chromosomal aberrations did not show a significant increase (*P* = 0.20). This differential biomarker response highlights varying sensitivity to WAG-induced genetic damage. Many studies reported exposure concentrations exceeding recommended occupational limits.

**Conclusion:**

This meta-analysis confirms that WAG exposure causes subclinical genotoxic damage, with biomarker patterns suggesting early DNA strand breaks and micronuclei formation are more sensitive indicators than chromosomal aberrations. These findings underscore the need for a precision prevention approach in occupational health: implementing enhanced safety measures and considering biomonitoring programs that utilize sensitive assays for early detection and individualized risk stratification among exposed personnel. Future research should investigate genetic and environmental modifiers of susceptibility to enable truly personalized risk assessment.

## 1 Introduction

Waste anesthetic gases (WAGs), including nitrous oxide (N2O) and halogenated anesthetics such as halothane, enflurane, isoflurane, sevoflurane, and desflurane, are routinely released into the operating room environment during the administration of anesthesia (1). These gases may escape from anesthetic breathing circuits, facemask connections, endotracheal tubes, or during system disconnections and induction, thereby contaminating the abient air even when scavenging and ventilation systems are in place (2, 3). Occupational exposure to WAGs affects a wide range of healthcare workers, including anesthesiologists, nurse anesthetists, surgeons, operating room nurses, and other perioperative personnel (4).

The health consequences of WAG exposure are diverse, ranging from short-term symptoms such as headache, nausea, fatigue, and dizziness to long-term complications including neurological impairment, hepatic and renal dysfunction, reproductive toxicity, and developmental abnormalities (5). Notably, the manifestation and severity of these effects may exhibit considerable inter-individual heterogeneity. Estimates from the Occupational Safety and Health Administration (OSHA) suggest that more than 200,000 healthcare professionals are at risk of developing occupational illnesses related to chronic WAG exposure (6, 7). Although the International Agency for Research on Cancer (IARC) classifies volatile anesthetics as Group 3 agents (not classifiable as carcinogenic to humans), concerns remain regarding their cumulative and subclinical biological effects, particularly in resource-limited settings where scavenging devices are absent or poorly maintained (8).

A growing body of evidence highlights that chronic WAG exposure may exert genotoxic effects by altering the genetic material of exposed individuals (9, 10). The potential impact of such exposure likely varies based on an individual’s genetic background and repair capacity, underscoring a need for a precision health approach. Studies have demonstrated DNA strand breaks, oxidative stress, and chromosomal aberrations in healthcare workers routinely exposed to these gases (11). Such genetic damage may underlie the increased risks of malignancy, infertility, spontaneous abortion, and congenital malformations observed in some occupational cohorts (12). The mechanisms proposed include oxidative DNA damage, interference with DNA repair pathways, and disruption of cell cycle regulation (13). Given the potential implications for occupational safety and public health, reliable biomonitoring techniques have been developed to assess genetic instability in exposed populations (1, 14). Among these, the comet assay (single-cell gel electrophoresis) provides a sensitive and reproducible method for detecting DNA strand breaks, while the micronucleus assay, particularly in exfoliated buccal epithelial cells, serves as a practical and inexpensive biomarker of mutagenicity (13, 15). These biomarkers are pivotal tools for a precision medicine framework in occupational health, enabling the move from population-level risk assessment toward individualized risk stratification and early detection. Together, these assays facilitate the identification of genotoxic hazards in healthcare workers exposed to WAGs (16).

Despite accumulating evidence, the literature remains heterogeneous in terms of study design, exposure assessment, biomarkers used, and populations studied. Therefore, a systematic review and meta-analysis is warranted to synthesize the available data and clarify the association between WAG exposure and genotoxic outcomes in healthcare workers. This systematic review and meta-analysis aims to quantify these genotoxic effects and evaluate the consistency of key biomarkers, thereby providing an evidence base to inform the development of precision prevention strategies, such as biomarker-guided monitoring and individualized risk assessment protocols, for occupational protection in healthcare settings.

## 2 Materials and methods

### 2.1 Study design

This systematic review and meta-analysis was conducted in accordance with the Preferred Reporting Items for Systematic Reviews and Meta-Analysis (PRISMA) 2020 guidelines (17). The study protocol was pre-designed prior to conducting the systematic review and meta-analysis, and all procedures were performed in accordance with this protocol.

### 2.2 Eligibility criteria

We included observational studies (cross-sectional, case-control, or cohort) that investigated healthcare workers occupationally exposed to waste anesthetic gases (WAGs) and assessed genotoxic effects using validated biomarkers. Eligible endpoints included DNA strand breaks (comet assay), micronucleus frequency, and chromosomal aberrations. Studies were required to include a control group of non-exposed participants, involve adult human subjects (>18 years), and report quantitative outcomes with sufficient statistical information for extraction. Exclusion criteria were: reviews, conference abstracts, editorials, case reports, animal or *in vitro* studies, and studies conducted in veterinary hospitals. Only full-text articles published in English were considered.

### 2.3 Search strategy

A comprehensive electronic search was performed across four databases: PubMed, Scopus, Google Scholar, and ScienceDirect. The search was last updated on September 1st, 2025, covering publications before August 2025. Boolean operators and Medical Subject Headings (MeSH) were applied, with the following search string: (“Anesthetic Gases” OR “waste anesthetic gases” OR “Nitrous Oxide” OR “halogenated anesthetics” OR “sevoflurane” OR “isoflurane” OR “desflurane”) AND (“Anesthesiologists” OR “Nurse anesthetists” OR “Operating room personnel” OR “Surgical staff”) AND (“DNA damage” OR “genotoxicity” OR “micronuclei” OR “chromosomal aberrations” OR “comet assay”) (Table 1). Additionally, reference lists of relevant articles were hand-searched to identify additional studies.

**TABLE 1:** Search strategy.

Search	Query	Results
Google Scholar	(“Anesthetic Gases” OR “waste anesthetic gases” OR “Nitrous Oxide” OR “halogen anesthetics” OR “halogen” OR “sevoflurane” OR “isoflurane” OR “desflurane”) AND “(Anesthetists” OR “Anesthesiologists” OR “Operating Room Nurse” OR” Anesthesiology resident” OR “Anesthetic Trainee” OR “Operating room personnel” OR “Operating room worker”) AND (“genetic damage” OR “genetic instability” OR “Genotoxic Effects”)	455
Pubmed	(“Anesthetic Gases” OR “waste anesthetic gases” OR “Nitrous Oxide” OR “halogen anesthetics” OR “halogen” OR “sevoflurane” OR “isoflurane” OR “desflurane”) AND (“Anesthetists” OR “Anesthesiologists” OR “Operating Room Nurse” OR “Anesthesiology resident” OR “Anesthetic Trainee” OR “Operating room personnel” OR “Operating room worker”) AND (“DNA Damage” OR “DNA Injury” OR “genetic damage” OR “genetic instability” OR “Genotoxic Effects”)	37
Science Direct	(“Anesthetic Gases” OR “waste anesthetic gases”) AND (“Anesthesiologists” OR “Operating Room Nurse” OR “Anesthesiology resident” OR “Operating room worker”) AND (“Genotoxic Effects”)	23
Scopus	(“Anesthetic Gases” OR “waste anesthetic gases” OR “Nitrous Oxide” OR “halogen anesthetics” OR “halogen” OR “sevoflurane” OR “isoflurane” OR “desflurane”) AND (“Anesthetists” OR “Anesthesiologists” OR “Operating Room Nurse” OR “Anesthesiology resident” OR “Anesthetic Trainee” OR “Operating room personnel” OR “Operating room worker”) AND (“DNA Damage” OR “DNA Injury” OR “genetic damage” OR “genetic instability”)	48

### 2.4 Study selection

All retrieved records were imported into Endnote X9 for deduplication and screening. Two independent reviewers (B.L. and H.Z.) screened titles and abstracts, with disagreements resolved by discussion or consultation with a third reviewer (Y.G.). Full-text articles meeting eligibility were further assessed against inclusion criteria.

### 2.5 Data extraction and quality assessment

A standardized data collection form was used to extract study information: author, year, country, study design, population characteristics (sample size, sex, age, occupation, smoking/alcohol history, body mass index), exposure duration, anesthetic gas type, and genotoxicity outcomes (DNA tail length, %DNA in tail, comet score, micronucleus frequency, chromosomal aberrations). The Newcastle-Ottawa Scale (NOS) was applied to evaluate risk of bias in case-control and cohort studies, while a modified NOS was used for cross-sectional studies (18, 19). Discrepancies were resolved by consensus. Studies were categorized as low, moderate, or high risk of bias.

### 2.6 Statistical analysis

Meta-analysis was conducted using Review Manager (RevMan) version 5.4.1 and Comprehensive Meta-Analysis (CMA) version 3.3. For each genotoxicity endpoint, mean differences (MD) with 95% confidence intervals (CI) were calculated. The standardized mean difference (SMD) approach was reserved for instances where different measurement scales were reported; otherwise, MD was utilized for consistency. If necessary, convert the median and interquartile range into mean and standard deviation.

Heterogeneity was assessed using the I^2^ statistic with thresholds of > 50% and *P* < 0.1 indicating substantial heterogeneity (20). Random-effects models were applied in the presence of significant heterogeneity; otherwise, fixed-effects models were used. Subgroup analyses were planned by biomarker type, gas type, and exposure duration where sufficient studies (*n* ≥ 3) were available for each category.

Potential publication bias was assessed through visual inspection of funnel plots and confirmed by Egger’s regression test for outcomes involving more than 8 included studies. A *P*-value < 0.05 was considered evidence of small-study effects. Sensitivity analyses were performed by excluding one study at a time or by removing identified outliers to evaluate the robustness of pooled estimates. Where sufficient data (≥10 studies per variable) were available, meta-regression analyses were planned to explore sources of heterogeneity, including study design and population characteristics.

## 3 Results

### 3.1 Study characteristics

**FIGURE 1:**
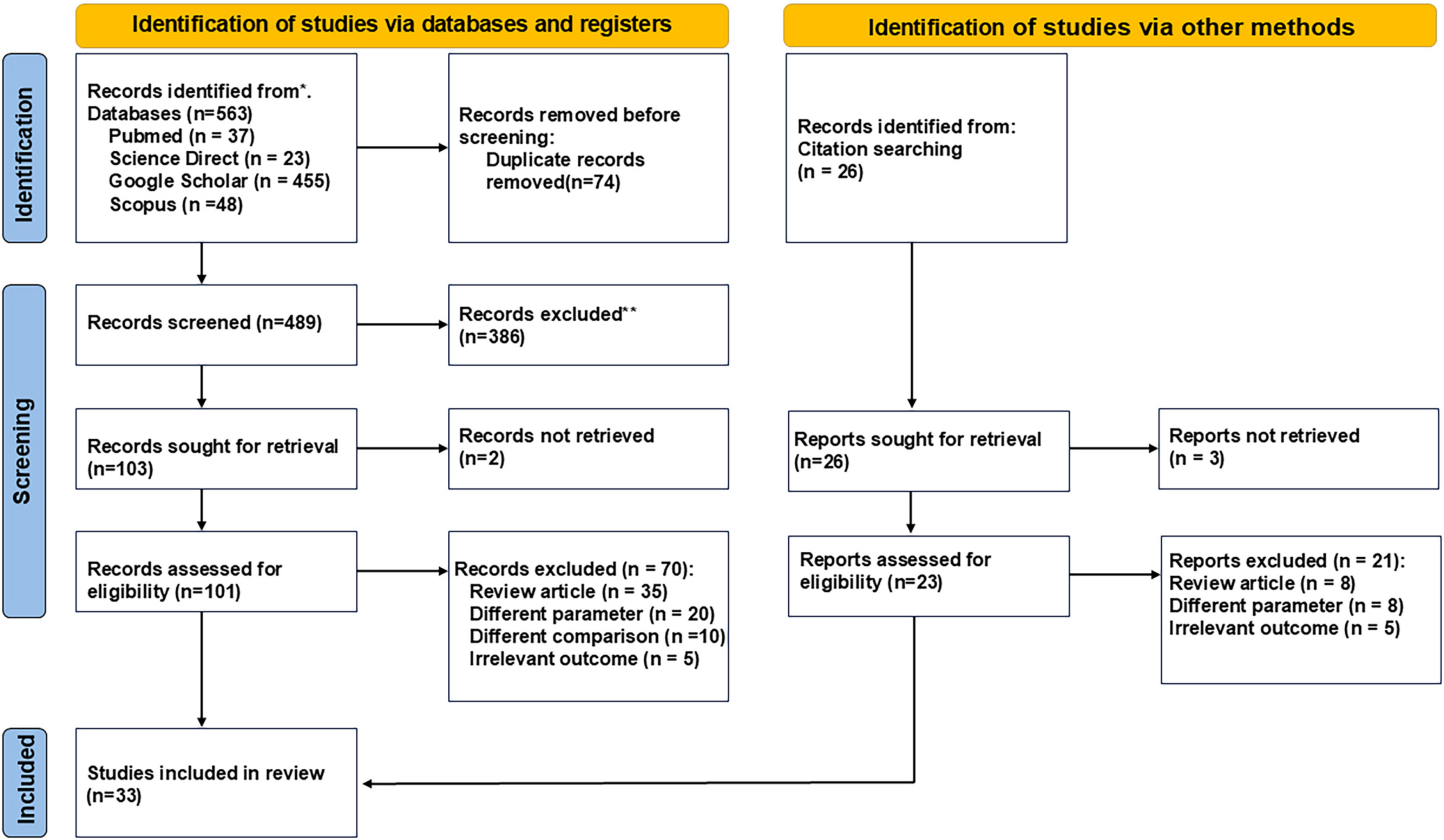
PRISMA flowchart of study selection.

The search strategy identified a total of 589 studies (563 from databases and 26 from citation searching). After removing duplicate records and screening, 489 records were assessed for eligibility. 103 records were sought for retrieval, and 101 records were evaluated in detail. Finally, 33 studies were included in the review (5, 13–16, 21–48) (Figure 1). Among the excluded records, 386 articles identified through database searching were deemed irrelevant or misaligned with the study criteria. Additionally, 72 reports from citation searching were excluded due to mismatched parameters or irrelevant outcomes, with 24 of these being excluded through this specific process. Most studies included were cross-sectional in design, and the screening phase highlighted multiple exclusions for reasons including irrelevant outcomes and review articles. Additionally, out of the 72 reports excluded during the retrieval process, 21 were review articles, and several others differed in parameters or outcomes. Characteristics of the study population can be seen in Table 2.

**TABLE 2:** Population’s characteristics.

Author	Study design	Country	Population (exposed/ control)	Physician proportion (exposed)	Age (exposed)^a^	Exposure period (year)^a^	Gas type	BMI	Gender (exposed, male/total)	Smoking (exposed, yes/total)	Alcohol (exposed, yes/total)
Braz et al. (58)	Cross-sectional	Brazil	43/43	43/43	34.6 ± 13.5	9.7 ± 12.2	I, N, S	25.4 ± 4.3	18/43	NA	NA
Scorza et al. (47)	Case-control	Brazil	19/19	19/19	26 ± 2	NA	D, I, N, S	24 ± 3	14/19	NA	NA
Silva et al. (16)	Cross-sectional	Brazil	100/93	74/100	33.96 ± 11.84	9.18 ± 10.86	H, N	25.26 ± 4.28	55/100	8/100	NA
Aldrieny et al. (21)	Cross-sectional	Egypt	26/13	NA	31.19 ± 3.06	10.89 ± 1.93	H, I	NA	15/26	2/26	NA
Hoerauf et al. (48)	Cross-sectional	Austria	10/10	10/10	32.80 ± 8.11	NA	H, N	NA	4/10	0/10	NA
Baysal et al. (22)	Case-control	Turkey	30/30	NA	33 ± 5	7 ± 4	D, H, I, N, S	25 ± 5	19/30	NA	NA
Bilban et al. (23)	Cross-sectional	Slovenia	153/153	153/153	NA	12.94 ± 6.52	H, I, N	NA	153/153	99/153	NA
Borayek et al. (24)	Cross-sectional	Egypt	32/32	0/32	34.9 ± 6.5	17.75 ± 5.3	I	NA	0/32	NA	NA
Braz et al. (25)	Cross-sectional	Brazil	30/30	30/30	28.5267 ± 1.61	3.06 ± 0.47	I, N, S	24.62	18/30	NA	NA
Braz et al. (13)	Cross-sectional	Brazil	31/32	NA	28.7 ± 1.9	3	I, N, S	24.6 ± 3.8	20/32	NA	NA
Braz et al. (15)	Cross-sectional	Brazil	40/40	40/40	39 ± 14.3	3.5	I,S	25.5 ± 3.2	26/40	NA	NA
Çakmak et al. (5)	Cross-sectional	Turkey	46/21	13/46	32.4 ± 5.7	NA	S	23.5 ± 3.2	9/46	21/46	4/46
Chandrasekhar et al. (14)	Case-control	India	45/45	19/45	38.76 ± 8.66	10.468 ± 4.70	D, E, H, I, N, S, SP	NA	25/46	20/46	15/46
de Araujo et al. (26)	Cross-sectional	Brazil	30/30	30/30	40.97 ± 11.25	13.83 ± 10.93	E, H, I, N, S,	NA	14/30	NA	NA
El-Ebiary et al. (27)	Cross-sectional	Egypt	40/20	23/40	39.6 ± 6.32	19.25 ± 2.36	H, I, N, S	NA	25/40	14/40	NA
Hua et al. (28)	Cross-sectional	China	68/82	NA	31.56 ±	8.29 ± 5.15	S	21.28	19/68	4/68	6/68
Izdes et al. (29)	Case-control	Turkey	40/40	0/40	36.8 ± 5.7	14.5 ± 6.6	D, I, N, S	NA	9/40	22/40	NA
Kargar Shouroki et al. (30)	Cross-sectional	Iran	60/60	10/60	36.17 ± 7.36	10.95 ± 5.58	I, N, S	20.75 ± 2.8	30/60	NA	NA
Kargar-Shouroki et al. (31)	Cross-sectional	Iran	45/45	45/45	37.73 ± 6.91	12.36 ± 6.3	N	NA	19/45	5/45	NA
Lewinska et al., (32)	Cross-sectional	Poland	46/28	0/46	42.9 ± 8.6	17.7 ± 10.1	I, N, S	NA	0/46	21/46	NA
Mušák et al. (33)	Cross-sectional	Czech Republic	76/76	41/76	36.89 ± 8.75	11.75 ± 9.35	NA	NA	15/76	23/76	NA
Neghab et al. (34)	Cross-sectional	Iran	60/60	NA	36.17 ± 7.36	10.95 ± 5.58	I, N, S	NA	30/60	NA	NA
Costa Paes et al. (35)	Cohort	Brazil	15/15	NA	27.9 ± 2.3	NA	I, N, S	25.5 ± 3.8	14/15	NA	NA
Rozgaj et al. (36)	Cross-sectional	Croatia	50/50	20/50	38.88 ± 7.59	12.96 ± 8.96	NA	NA	12/50	16/50	NA
Santovito et al. (37)	Cross-sectional	Italy	21/21	21/21	35.524	8.619 ± 4.364	NA	NA	15/21	NA	NA
Shaker et al. (38)	Cross-sectional	Egypt	27/18	0/27	33.7 ± 7	15 ± 6.7	D, I, N, S	NA	0/27	0/27	NA
Silva et al. (39)	Cross-sectional	Brazil	100/93	NA	34.2 ± 11.8	NA	I, N, S	25.5 ± 4.3	55/100	8/100	70/100
Souza et al. (40)	Cross-sectional	Brazil	30/30	30/30	42 ± 15.9	NA	D, I, N, S	26.1 ± 3.3	20/30	NA	NA
Souza et al. (41)	Cross-sectional	Brazil	30/30	30/30	NA	NA	H, N	26 ± 3	20/30	NA	NA
Szyfter et al. (42)	Cross-sectional	Poland	100/100	26/100	NA	NA	NA	NA	15/100	24/100	NA
Wiesner et al. (43)	Cross-sectional	Germany	14/14	14/14	32 ± 5	NA	S	NA	8/14	4/14	NA
Wron’ska-Nofer et al. (44)	Cross-sectional	Poland	84/83	29/84	40.73	15.77	I, N, S	NA	29/84	39/84	NA
Wronska-Nofer et al. (45)	Cross-sectional	Poland	36/36	0/36	NA	NA	I, N, S	NA	0/36	NA	NA

Results presented in

*^a^*Mean (range). NA, Data Not Available, D, Desflurane; E, Enflurane; H, Halothane; I, Isoflurane; N, Nitrous oxide; S, Sevoflurane; SP, Sodium pentothal.

There are five types of gases reported across the investigations in the operating room environment, including Nitrous Oxide (17 studies), Isoflurane (12 studies), Sevoflurane (7 studies), Desflurane (4 studies), and Halothane (2 studies). Regrettably, some investigations found that the regular exposure limit for nitrous oxide (25 ppm time-weighted average/TWA) was exceeded. For example, Lewinska et al. (32) and Neghab et al. (34) reported values up to 850.92 ppm. Similarly, the exposure limit for halogenated anesthetics (2 ppm) was exceeded in Neghab et al. (34), with values reaching up to 16.4 ppm for Desflurane (34). Meanwhile, several studies did not report any information on WAGs concentration, including Wiesner et al. (43) and Wron’ska-Nofer et al. (44) WAGs concentration data are listed in Table 3.

**TABLE 3:** Concentrations (ppm) of WAGs in operating rooms.

Author	N_2_O	Isoflurane	Sevoflurane	Desflurane	Halothane
NIOSH recommended exposure limits	25	2	2	2	2
Braz et al. (58)	140 ± 19.3	8 ± 1.6	10 ± 6.3	–	–
Scorza et al. (47)	154 ± 20	5.6 ± 3.7	9.9 ± 7.1	15.3 ± 6.8	–
Silva et al. (16)	165 ± 15	–	–	–	8 ± 6
Bilban et al. (23)	0–100^b^	0–10^b^	–	–	0–10
Hoerauf et al. (48)	295.2 (0.5–199.53) ^a^	5.3 (0.1–125.9) ^a^	–	–	–
Braz et al. (25)	155 ± 138	5.1 ± 4.2	9.8 ± 9.0	–	–
Braz et al. (13)	180 (61–350)^a^	5.3 (0.3–17.8)^a^	9.7 (1.0–34.1)^a^	–	–
Braz et al. (15)	–	1.25 ± 0.61^a^	1.74 ± 0.73^a^	–	–
Çakmak et al. (5)	–	–	0.427 (0.32–0.58)^a^	–	–
Hua et al. (28)	–	–	1.11 ± 0.65	–	–
Lewińska et al. (32) *	7.78–1282.13	–	–	–	–
Neghab et al. (34)	850.92 (10–3895)^a^	2.4 (0.49–4.15)^a^	0.18 (0.01–0.59)^a^	–	–
Kargar-Shouroki et al. (31)	450.27 ± 327.44^a^	–	–	–	–
Silva et al. (39)	165 ± 15	7 ± 5	9 ± 7	–	–
Souza et al. (40)	150.3 ± 135.7	5.5 ± 4.4	7.7 ± 8.7	16.4 ± 6.0	–
Souza et al. (41)	150 ± 136	–	–	–	10 ± 6.4
Wiesner et al. (43)	–	–	0.2 (0.08–2.24)^c^	–	–
Wron’ska-Nofer et al. (44) *	244.43 (19.89–834.39)^a^	0.689 (0.066–1.855)^a^	0.574 (0.05–1.83)^a^	–	–
Wron’ska-Nofer et al. (45)*	102.77–834.39 ^b^	0.053–1.988 ^b^	0.061–1.711 ^b^	–	–

*Value presented as the conversion from mg/m^3^ using the formula: Concentration (ppm) = 24.45 × concentration (mg/m^3^)/molecularweight. Data was presented in mean ± standard deviation except stated otherwise (^a^ Mean (range) ^b^ Range, ^c^ Median (range). Data was compiled only from studies which stated the gas concentration explicitly.

### 3.2 Quality assessment

The Newcastle-Ottawa Scale (NOS) was used to evaluate the risk of bias across all included studies. The NOS instrument was modified to fit the appraisal of cross-sectional designs. In total, 33 studies were assessed, with overall scores ranging from 4 to 8. Of these, 13 studies (39.4%) were rated as having a low risk of bias (score 7–8), 18 studies (54.5%) were rated as moderate risk (score 5–6), and 2 studies (6.1%) were judged as high risk (score 4). The mean NOS score for the included studies was 6.12 ± 1.07, reflecting an overall moderate-to-low risk of bias across the evidence base. Table 4 provides a detailed summary of the risk of bias assessment.

**TABLE 4:** Risk of bias analysis.

Cross sectional	Selection	Comparability	Outcome	Total score	Interpretation (risk of bias)
Braz et al. (58)	3	0	2	5	Moderate
Scorza et al. (47)	4	0	1	5	Moderate
Silva et al. (16)	4	0	1	5	Moderate
Hoerauf et al. (48)	2	0	2	4	High
Aldrieny et al. (21)	3	0	2	5	Moderate
Bilban et al. (23)	4	0	1	5	Moderate
Borayek et al. (24)	4	0	1	5	Moderate
Braz et al. (25)	4	0	2	6	Moderate
Braz et al. (13)	4	0	2	6	Moderate
Braz et al. (15)	4	1	2	7	Low
Çakmak et al. (5)	4	2	2	8	Low
de Araujo et al. (26)	4	1	1	6	Moderate
El-Ebiary et al. (27)	4	0	1	5	Moderate
Hua et al. (28)	4	0	1	5	Moderate
Kargar Shourouki et al. (30)	4	2	2	8	Low
Kargar-Shourouki et al. (31)	4	1	1	6	Moderate
Lewińska et al. (32)	4	2	2	7	Low
Mušák et al. (33)	4	0	1	5	Moderate
Neghab et al. (34)	4	2	2	8	Low
Rozgaj et al. (36)	4	0	2	6	Moderate
Santovito et al. (37)	4	0	2	6	Moderate
Shaker et al. (38)	4	0	2	5	Moderate
Silva et al. (39)	4	2	2	8	Low
Souza et al. (40)	4	1	1	6	Moderate
Souza et al. (41)	4	2	2	8	Low
Szyfter et al. (42)	4	2	2	8	Low
Wiesner et al. (43)	4	2	2	8	Low
Wron’ska-Nofer et al. (44)	4	0	1	5	Moderate
Wronska-Nofer et al. (45)	3	2	2	7	Low
Baysal et al. (22)	2	0	2	4	High
Chandrasekhar et al. (14)	2	2	3	7	Low
Izdes et al. (29)	2	1	3	6	Moderate
Costa Paes et al. (35)	3	0	3	6	Moderate

### 3.3 Meta-analysis on impact of anesthetic gas exposure to comet assay, micronuclei formation, and chromosomal aberration

**FIGURE 2:**
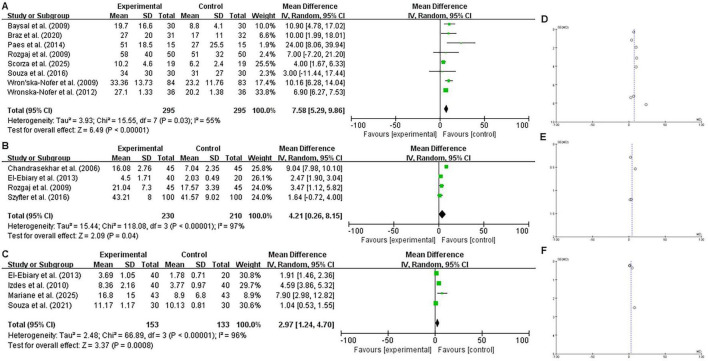
Primary meta-analysis of the impact of occupational WAGs exposure on DNA damage indicators (Full Dataset). Forest plots **(A–C)** illustrate the pooled mean differences (MD) with 95% confidence intervals (CI) for: **(A)** DNA damage score (arbitrary units, ranging from 0 to 400), **(B)** comet tail length (measured in μm), and **(C)** percentage of DNA in the comet tail (examined via computerized image evaluation). These plots represent the primary analysis using a random-effects model (*I*^2^ > 50%) to account for baseline heterogeneity across all eligible studies. The green squares represent the weight of individual studies, and the black diamonds signify the overall pooled effect. Corresponding funnel plots **(D–F)** are provided to assess potential publication bias for each respective outcome. A symmetrical distribution in the funnel plots generally indicates the absence of significant small-study effects.

**FIGURE 3:**
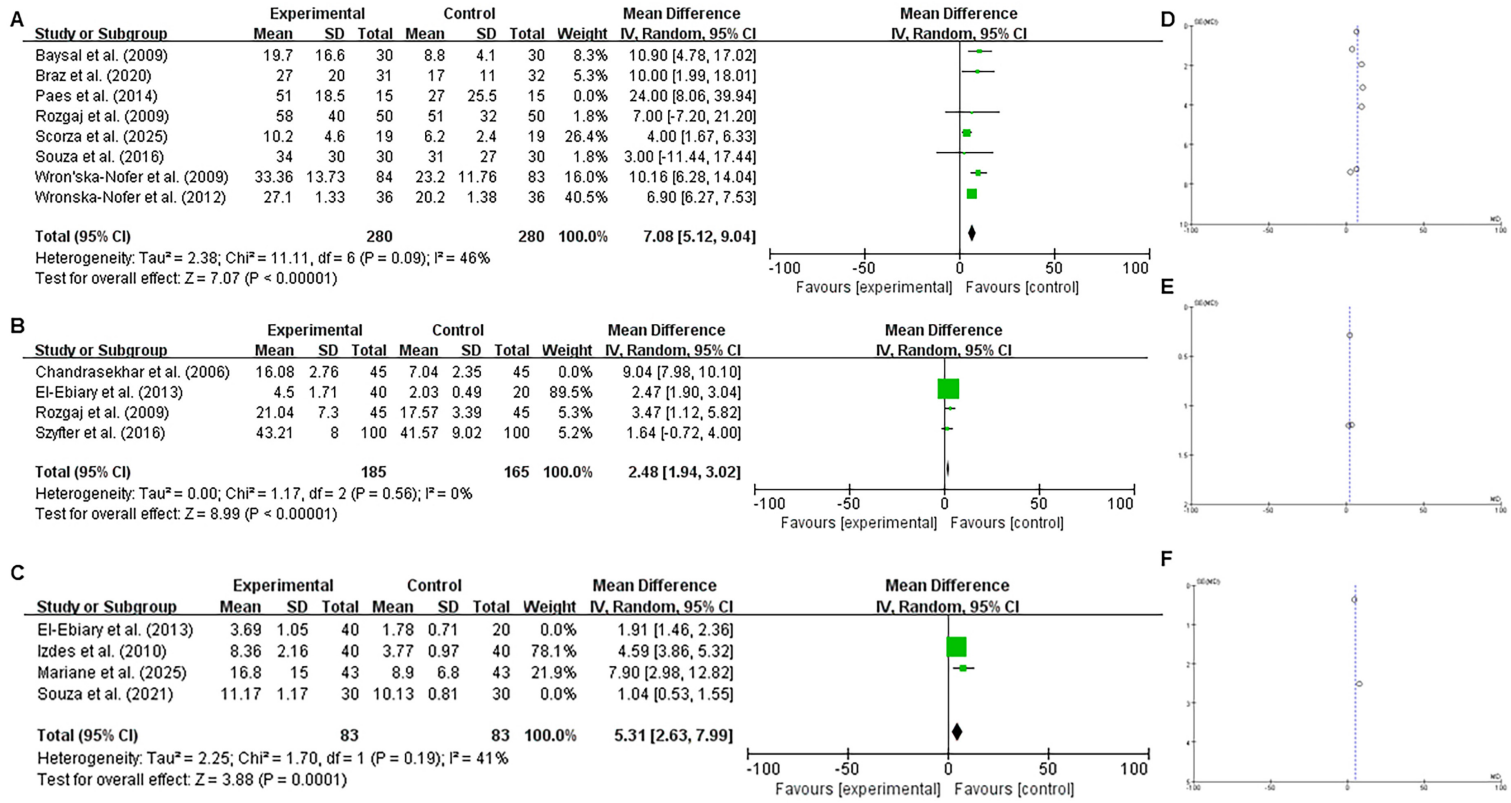
Sensitivity analysis of DNA damage indicators following the exclusion of heterogeneous studies. Forest plots **(A–C)** display the refined pooled estimates for: **(A)** DNA damage score, **(B)** comet tail length, and **(C)** % tail DNA after performing sensitivity analysis. This analysis was conducted by systematically removing studies identified as primary sources of statistical heterogeneity to evaluate the robustness of the initial findings. Note the reduction in the I^2^ statistic and the narrowed 95% CI compared to the primary analysis, indicating improved consistency of the evidence. Funnel plots **(D–F)** represent the publication bias assessment for the remaining study cohort, ensuring that the filtered evidence base remains reliable and representative.

**FIGURE 4:**
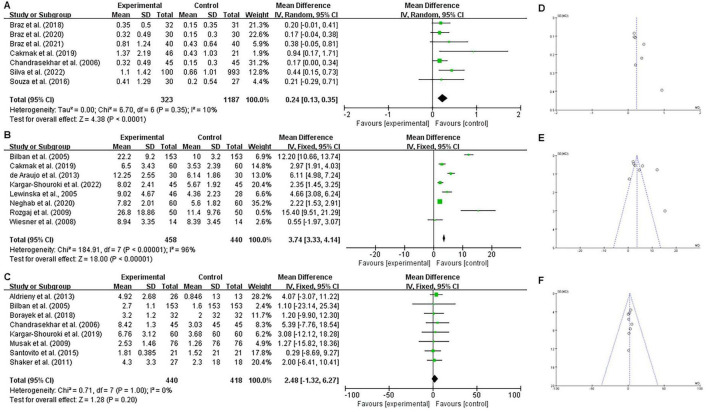
Effect of occupational exposure to waste anesthetic gases (WAGs) on cytogenetic damage. **(A)** Buccal micronuclei formation, **(B)** Lymphocyte micronuclei formation, and **(C)** Total chromosomal aberrations, presented as mean differences with 95% confidence intervals (forest plots). Data in **(A,B)** are expressed per 1,000 cells, while data in **(C)** are expressed per 100 cells. Funnel plots for the same outcomes are shown in **(D–F)**, corresponding to **(A–C)**, respectively.

In the primary meta-analysis involving all eligible studies, exposed individuals showed significant DNA damage compared to non-exposed controls across all comet assay parameters: DNA damage score (MD = 7.58, 95% CI = 5.29–9.86; *P* < 0.0001), tail length (MD = 4.21, 95% CI = 0.26–8.15; *P* = 0.0400), and percentage of DNA in the comet tail (MD = 2.97, 95% CI = 1.24–4.70; *P* = 0.0008) (Figures 2A–C). Due to the substantial heterogeneity observed (*I*^2^ > 50%, *P* < 0.1), we performed sensitivity analyses by systematically excluding studies that contributed most significantly to the variance. The significant differences persisted after the removal of these heterogeneous studies, with pooled MDs of 7.08 (95% CI = 5.12–9.04; *P* < 0.0001) for DNA damage score, 2.48 (95% CI = 1.94–3.02; *P* < 0.0001) for tail length, and 5.31 (95% CI = 2.63–7.99; *P* = 0.0001) for % tail DNA. These results confirm the robustness of our initial findings (Figures 3A–C). Regarding cytogenetic damage (Figures 4A–C), similar significant trends were observed for buccal micronuclei (MD = 0.24, 95% CI = 0.13–0.35; *P* < 0.0001) and lymphocyte micronuclei (MD = 3.74, 95% CI = 3.33–4.14; *P* < 0.0001), while total chromosomal aberration showed no significant difference (*P* = 0.20).

Notably, because the number of studies for specific gas types and exposure durations did not meet our pre-specified threshold (*n* ≥ 10 per variable), meta-regression was not performed, and planned subgroup analyses were limited to those with sufficient comparative data.

### 3.4 Publication bias

Publication bias was assessed using funnel plots. From visual inspection (Figures 2D–F, 3D–F, 4D–F), only buccal micronucleus formation showed an asymmetric distribution of the pooled results, suggesting the possibility of publication bias. In contrast, the DNA damage indicators—including DNA damage score (comet assay arbitrary unit), comet tail length, % tail DNA, lymphocyte micronuclei, and total chromosomal aberrations—did not reveal evidence of publication bias.

## 4 Discussion

This systematic review and meta-analysis demonstrates that occupational exposure to waste anesthetic gases (WAGs) is associated with measurable genotoxic effects in healthcare workers. Across 33 studies, exposed personnel exhibited higher DNA damage by comet assay—reflected in greater arbitrary damage scores (MD = 7.58), longer tail length (MD = 4.21), and higher %tail DNA (MD = 2.97)—and increased micronucleus formation in both buccal cells (MD = 0.24) and lymphocytes (MD = 3.74) compared with non-exposed controls. These associations persisted after sensitivity analyses that removed heterogeneous studies, indicating that the signal is robust to study-level variability. By contrast, total chromosomal aberrations were not significantly elevated (MD = 2.48; *P* = 0.20), suggesting that WAG-related damage is more readily captured by assays sensitive to primary DNA strand breaks and short-lived genomic lesions than by cytogenetic endpoints that often reflect more advanced or clonally fixed alterations. This differential sensitivity among biomarkers has direct implications for a precision medicine approach in occupational health. Specifically, the comet and micronucleus assays, due to their sensitivity to early and prevalent damage, may serve as ideal tools for early detection and individual risk stratification in routine biomonitoring programs. In contrast, the assessment of chromosomal aberrations might be reserved for evaluating long-term cumulative risk in potentially highly susceptible subpopulations.

The divergence between comet/micronucleus findings and conventional chromosomal aberrations likely stems from biological timing and assay sensitivity (49). Comet parameters detect transient single- and double-strand breaks and oxidative lesions that may precede stable cytogenetic changes, whereas chromosomal aberrations require either persistent damage beyond cellular repair capacity or prolonged exposure sufficient for mis-segregation and structural rearrangements to accrue (50, 51). Inter-individual differences in DNA repair efficiency, antioxidant defenses, and genetic susceptibility could therefore yield strong effects on comet and micronucleus outcomes without a parallel rise in overt cytogenetic abnormalities. Mechanistically, the pattern we observed aligns with oxidative stress, impaired base-excision or mismatch repair, and cell-cycle checkpoint perturbation that have been proposed for nitrous oxide and halogenated agents (52–54). These mechanistic insights underscore the potential for variable individual susceptibility, a core tenet of precision medicine. Future research identifying genetic or epigenetic modifiers of these pathways could enable the prediction of individual risk.

From an occupational health perspective, these results carry practical implications. Although WAGs are categorized by IARC as Group 3 (not classifiable as carcinogenic to humans) (55, 56), our pooled estimates indicate consistent subclinical genotoxic changes in exposed cohorts. Importantly, several included investigations documented operating-room concentrations that exceeded recommended limits, with nitrous oxide levels reported up to ∼851 ppm and halogenated agent concentrations (e.g., desflurane) up to ∼16.4 ppm—far above typical 25 ppm (TWA) and 2 ppm guidance, respectively. Such exceedances were more common where scavenging systems were absent, outdated, or poorly maintained, or where room ventilation and work practices were suboptimal. These observations reinforce the need to prioritize engineering controls (effective active scavenging, adequate air exchanges, leak testing of circuits), administrative measures (procedural checklists, staff training, maintenance logs, and exposure monitoring), and, where appropriate, task redesign to minimize open-system steps that elevate ambient concentrations (24, 35, 48, 52, 56, 57). Beyond these universal controls, our findings advocate for a precision prevention strategy. The integration of sensitive biomonitoring into health surveillance programs could allow for the identification of individuals or teams exhibiting higher-than-expected genotoxic responses, prompting targeted interventions such as enhanced personal protection, environmental re-assessment, or individual susceptibility screening. This shifts the paradigm from a one-size-fits-all approach to a more efficient, biomarker-informed resource allocation for worker protection.

The findings of this meta-analysis provide a compelling rationale for integrating precision medicine principles into occupational health management of WAG exposure. The core objective of precision medicine, to tailor preventive and therapeutic strategies based on individual variability in risk, aligns perfectly with the observed heterogeneity in genotoxic responses. Our results indicate that while exposure is a population-level hazard, the measurable biological effect varies, potentially influenced by individual genetic makeup, repair capacity, and other endogenous factors. Therefore, moving beyond generic exposure limits, a precision framework would involve: (i) Using sensitive biomarkers like the comet assay to identify “high-response” individuals who may benefit from more frequent monitoring or enhanced protective measures, even at exposure levels deemed acceptable for the general population. (ii) Actively investigating the sources of inter-individual variation. Future studies must prioritize collecting data on genetic polymorphisms, epigenetic profiles, and lifestyle factors to build predictive models of susceptibility. (iii) Developing interventions based on an individual’s specific risk profile, which could range from personalized protective equipment assignments to tailored work schedule rotations in high-exposure areas. This approach not only enhances protection for the most vulnerable but also improves the overall efficiency of occupational health resources.

This meta-analysis possesses several methodological strengths, most notably a pre-specified protocol strictly aligned with PRISMA guidelines. By integrating multiple complementary genotoxic biomarkers and employing a robust quantitative pooling strategy supplemented by sensitivity analyses, this study provides a comprehensive synthesis of the current evidence base. Such a multi-faceted approach enhances the internal validity of our findings and allows for a more nuanced evaluation of genomic instability associated with anesthetic gas exposure compared to single-endpoint assessments. This synthesis directly informs the evidence base needed to develop stratified or personalized risk models in the future.

However, several inherent limitations necessitate a cautious interpretation of the observed associations. The predominance of cross-sectional designs among the included studies precludes definitive causal inference and increases susceptibility to the “healthy-worker effect” and reverse causation. Furthermore, substantial heterogeneity in exposure assessment—ranging from direct environmental monitoring to reliance on surrogate markers like job titles—introduces the potential for non-differential misclassification, which may bias estimates toward the null. Additionally, our literature search was restricted to four electronic databases (PubMed, Scopus, Google Scholar, and ScienceDirect). Although we supplemented this with manual searches of reference lists, the exclusion of other databases such as Embase or Web of Science may have resulted in the omission of some relevant studies. While the overall risk of bias remained moderate-to-low, inconsistent adjustment for critical confounders such as smoking, BMI, and ionizing radiation co-exposures suggests that residual confounding cannot be entirely dismissed. The sparse data available for specific anesthetic agents and demographic subgroups limited our capacity for robust meta-regression, while funnel plot asymmetry for buccal micronucleus outcomes hints at potential small-study effects. Finally, the scarcity of longitudinal data linking these intermediate biomarkers to clinical endpoints, such as malignancy or reproductive adversity, underscores the need for future prospective cohorts.

## 5 Conclusion

This meta-analysis demonstrates that occupational exposure to WAGs significantly increases DNA damage and micronucleus formation in healthcare workers. While chromosomal aberrations were not significantly elevated, the findings highlight the genotoxic risks of WAG exposure. The distinct response patterns across different biomarkers underscore the importance of assay selection for biomonitoring and reflect underlying biological variability. To protect workers’ health, a dual approach is recommended: first, the universal implementation of enhanced engineering and administrative controls; second, and more innovatively, the adoption of a precision health strategy. This strategy involves utilizing sensitive biomarkers for early detection and individual risk stratification, and calls for future research to identify the genetic and molecular factors governing inter-individual susceptibility. Ultimately, moving from a uniform protection standard toward personalized risk assessment and targeted interventions represents the most promising avenue for advancing occupational health in this field.

## Data Availability

The original contributions presented in the study are included in the article/supplementary material, further inquiries can be directed to the corresponding author.
